# Developmental Toxicity of Carbon Quantum Dots to the Embryos/Larvae of Rare Minnow (*Gobiocypris rarus*)

**DOI:** 10.1155/2016/4016402

**Published:** 2016-10-31

**Authors:** Yuan-Yuan Xiao, Li Liu, Yao Chen, Yu-Lian Zeng, Ming-Zhi Liu, Li Jin

**Affiliations:** Key Laboratory of Freshwater Fish Reproduction and Development, Key Laboratory of Aquatic Science of Chongqing, School of Life Sciences, Southwest University, Ministry of Education, Chongqing 400715, China

## Abstract

The toxic effects of CDs on rare minnow (*Gobiocypris rarus*) embryos at different developmental stages were investigated. The results showed that rare minnow embryos had decreased spontaneous movements, body length, increased heart rate, pericardial edema, yolk sac edema, tail/spinal curvature, various morphological malformations, and decreased hatching rate. Biochemical analysis showed the CDs exposure significantly inhibited the activity of Na^+^/K^+^-ATPase and Ca^2+^-ATPase and increased the MDA contents and the activity of SOD, CAT, and GPX. Further examination suggested that the CDs exposure induced serious embryonic cellular DNA damage. Moreover, the CDs exposure induced upregulation of development related genes (*Wnt8a* and* Mstn*) along with the downregulation of* Vezf1*. Overall, the present study revealed that the CDs exposure has significant development toxicity on rare minnow embryos/larvae. Mechanistically, this toxicity might result from the pressure of induced oxidative stress coordinate with the dysregulated development related gene expression mediated by the CDs exposure.

## 1. Introduction

Carbon quantum dots (CDs) denote a class of less than 10 nm sized nanoparticles composed of carbon, hydrogen, oxygen, nitrogen, and other elements [1]. Bearing the advantage as higher thermostability, photostability, quantum yield, and lower release rate of heavy metal ions than the traditional semiconductor quantum dots [2], CDs have been proposed broadly to the potential application in analytical chemistry, biological probing and imaging, drug delivery, and so forth [3–5]. Further, with the advent of nanotechnology, CDs are becoming more prevalent in the environment. This raises increasing public concerns about biosafety and the potential health risks of long term exposure to CDs. Previous study has shown that concentration of the TiO_2_ in surface waters is 21 ng/L and the C_60_ in sewage is 4 ng/L, which could increase to 35 mg/L through the suspension process in the natural water system [6]. Further, a series of studies has shown the nanomaterials including CdSe/ZnS and TiO_2_ could induce the fishes embryos' developmental defect such as embryonic pericardial edema, yolk sac edema, and tail malformation [7], suggesting the potential risky impact of CDs on the natural water ecosystem. Thus, the biological and environmental toxicity of CDs gradually attracted great research interest which is required for the safety evaluation for further application.

However, only limited studies regarding this issue have been reported while the comprehensive toxic effects and the exact underlying mechanism are still not clear. In 16 HBE cells, high concentration of CDs treatment results in oxidative damage and decreased proliferation rate. Besides, CDs could form a complex with BSA leading to the structural alteration of some functional proteins [8]. In animal models, when CDs were administrated through the tail vein injection in rats, the oxidative damage and the disrupted immunologic balance were observed in the target organs which eventually induced the compensable pathological lesions [9].

Here we report a biosafety research of CDs on rare minnow (*Gobiocypris rarus*), an endemic cyprinid fish in China [10]. With serious features such as short life cycle, high fertilization rates, and hatching rates, rare minnow has been regarded as one of the suitable aquatic organisms for the biological toxicity assessment [11].

In our study, we have investigated the developmental toxicity of the CDs on the embryos/larvae of rare minnow. Our data would not only enrich the scientific understanding for the biocompatibility and risk assessment of CDs, but also promote the early diagnosis of potential pollution in fishery water area introduced by nanomaterials.

## 2. Materials and Methods

### 2.1. CDs

CDs (W-900-440) were purchased from Beida Jubang Science & Technology Co. Ltd. (Beijing, China). Monodispersed CDs were 2–6 nm sized and prepared as 10 mg/mL (water solution). The fluorescence emission wavelength of the CDs is 440 nm.

### 2.2. Fish Husbandry and Embryo Collection

Sexually matured and healthy rare minnow were selected as parent fish. Following long term acclimation (more than 4 weeks) in the circulating water system with the sex ratio as 1 : 1, the spawning time points were set to 20:00 daily through the circadian rhythm manipulation. Once the sexual chasing behavior of the parent fishes was observed, the fertilized eggs were collected followed by artificial insemination (fertilization rate > 95%). The parent fishes were fed three times daily to visual satiation (twice with commercial granular food and once with fairy shrimp). Culture condition was set as 25 ± 1°C with the daily photoperiod cycle as 12 : 12 (light : dark).

### 2.3. Embryo Toxicity Assay

The toxicity assay was designed according to the established protocol [12]. Briefly, the rare minnow embryos were collected immediately after fertilization; at blastocyst stage, normal embryos were selected and introduced individually into 24-well plates filled with 1 mL/well daily renewed solutions or controls per well. The plates were incubated at 25 ± 1°C in water bath for 96 h with the photoperiod of 12 : 12 h (light : dark). The toxicity effects of CDs on the subsequent embryonic development of rare minnow were evaluated by exposing fertilized eggs to a range of concentrations (0, 1, 5, 10, 20, 40, and 80 mg/L). The standard dilution solution served as the control. Three wells for concentration groups and three replicates were set for the tests, with 20 embryos per replicate. In our study, embryos/larvae from the five early embryonic development stages (12 hpf, 24 hpf, 48 hpf, 72 hpf, and 96 hpf) were sampled and analyzed.

### 2.4. Microexamination

The developmental parameters were monitored and documented every six hours between 12 hpf (hours after fertilization) and 96 hpf. Toxicological endpoints were determined by the observation through microscopy (Nikon SMZ 25, Japan). Between 12 hpf and 96 hpf stage, the hatching rate, the mortality rate, and the malformation rate were calculated. The spontaneous movement frequency at 36 hpf stage and the heart rate at 60 hpf stage were calculated, respectively, as well. The body length (mm), the area of pericardial edema (*μ*m^2^), area of sac-yolk edema (*μ*m^2^), and SV-BA distance (*μ*m) at 96 hpf stage were photographed with a digital camera and measured from these digital images using Image pro plus 6.0 software (Media Cybernetics, America).

### 2.5. Measurement of Antioxidant Enzymes, Na^+^/K^+^-ATPase, Ca^2+^-ATPase Activity, and Malondialdehyde (MDA) Content

20 embryos/larvae were collected from each group. Each sample was defrosted and homogenized on ice with 0.65% ice-cold saline. Following centrifuging at 2500 ×g at 4°C for 10 min, the supernatants were collected and subject to the measurement of the MDA content and the activity of the antioxidant enzymes, Na^+^/K^+^-ATPase, Ca^2+^-ATPase based on the kits manual (Nanjing Jiancheng Bioengineering Institute, Nanjing, China).

### 2.6. Comet Assay

20 embryos/larvae were collected from each group. Samples were completely digested in 37°C water bath with 0.25% trypsin. Following centrifuging at 2500 ×g for 10 min and the supplementation with moderate amount of PBS (pH 7.4), the single-cell suspension (10^4^–10^6^/mL) was prepared and subject to the single-cell gel electrophoresis (SGCE). For each treatment group, about 100 cells per slide were randomly scored with an image analysis through fluorescent microscopy. The comet images were analyzed by the auto comet image analysis system [13]. The DNA damage of the samples was evaluated by comet tail length, percentage of total DNA in the tail, tail moment, and olive tail moment.

### 2.7. Gene Expression Analysis

According to the gene sequences in GenBank, primers were designed by the primer 5.0 software (Table 1) and synthesized by biotechnology company (Invitrogen). The total RNA of tested rare minnow embryos/larvae was extracted using the RNAiso Plus (Takara, Kyoto, Japan). The total RNA contents were determined by measuring the absorbance at 260 nm and the RNA quality was verified by the ratio of the absorbance at 260/280 nm (range within 1.8~2.0). The first-strand cDNA synthesis was performed by using PrimeScript ®RT reagent Kit with gDNA Eraser (Takara, Kyoto, Japan) following the manufacturer's instruction. The qPCR results were calculated using 2^−ΔΔCT^ method [14].

### 2.8. Statistical Analysis

Statistical analysis was conducted with the one-way ANOVA or* t*-test depending on heterogeneity of variance (SPSS 17.0). Following the one-way ANOVA analysis, LSD multiple range test was employed for the evaluation between control and treated groups; otherwise* t*-*test* was performed between control and treated groups because of heterogeneity of variance. The differences were considered significant at* p* < 0.05 and extremely significant at* p* < 0.01.

## 3. Results

### 3.1. Morphological and Behavioral Analysis of Embryo Development of Rare Minnow

**Figure 1:**
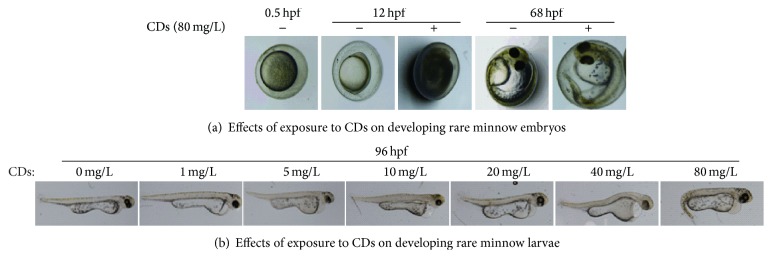
Effects of exposure to CDs on developing rare minnow embryos/larvae.

The toxicity effect of CDs on rare minnow embryos/larvae at different developmental stage was investigated in a concentration-dependent manner. As shown in Figure 1(a), in the stage of 12 hpf, no obvious developmental defects were observed in lower concentration groups (1, 5, 10, and 20 mg/L) compared with the control group. In higher concentration groups (40 mg/L and 80 mg/L), embryos yolk turbidity/agglutination was observed. Moreover, longer exposure time increased the malformation rate indicated by the shorter body length, tail/spinal curvature and pericardial edema, and yolk sac edema of the rare minnow embryos/larvae (Figure 1(b)).

**Figure 2:**
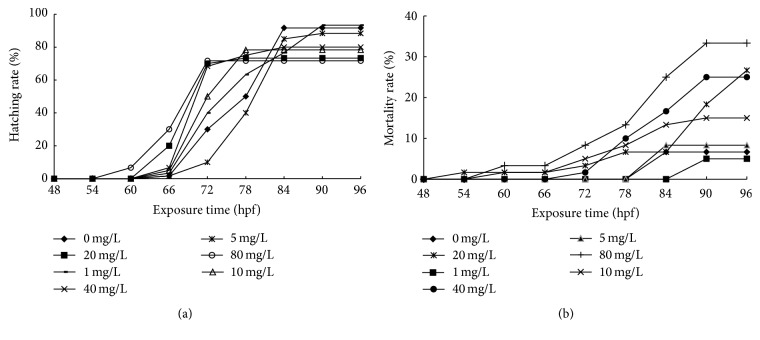
The hatching rate and mortality rate of rare minnow embryos exposed to different CDs concentration. The hatching rate (a) and mortality rate (b) for rare minnow embryos exposed to different CDs concentration. Triplicates were set for the tests, with 20 embryos/larvae each time.

The effects of CDs on hatching rate and mortality rate in embryos of rare minnow were tested. As shown in Figure 2, compared with the control group, the highest CDs concentration resulted in accelerated hatching. The hatched larvae were firstly observed at the stage of 54~60 hpf in the highest concentration group while they were detected at the stage of 60~66 hpf in control group. The hatching rate decreased significantly in response to the CDs treatment (Figure 2(a)). When the exposure time was extended to 96 hpf, most of unhatched embryos died.

Consistently, the increased mortality rate was observed in a concentration-dependent manner following the exposure to CDs at the stage of 96 hpf. It is interesting to note that no significant change among all concentration group treatments was detected before 72 hpf in contrast to the marked elevation of the mortality rate after 72 hpf (Figure 2(b)). It suggested that 72 hpf stage might be important for the fate determination of the rare minnow embryo development in response to the environmental stress introduced by the CDs.

The spontaneous embryonic movement at 36 hpf stage decreased in a concentration-dependent manner after the exposure to CDs. In the 10, 20, and 40 mg/L groups, the spontaneous embryonic movements decreased significantly against control group (*p* < 0.01). At the 60 hpf stage, the embryos heart rate in control group was 67.00 ± 2.45 beats per 30 seconds (mean ± SD) (Table 2). Embryos heart rates increased significantly in 20 mg/L group (*p* < 0.01). In the 40 and 80 mg/L group, heart rates just increased slightly. The malformation rates were increased in a concentration-dependent manner after the CDs exposure at the stage of 96 hpf. In 10~80 mg/L concentration group, the malformation rate was significantly increased compared with that in the control group (*p* < 0.01).

We also investigated the effects of CDs on hearts development of rare minnow embryos. In the 1 and 5 mg/L concentration group, compared with control, no significantly changed SV-BA distance was detected (*p* < 0.05) while, in the 10~80 mg/L concentration groups, the SV-BA distance was significantly increased (*p* < 0.01). The significant enlarged area of pericardial edema and sac-yolk edema appeared, respectively, in proportion to the increasing CDs concentration. In groups with concentration higher than 10 mg/L, the areas of pericardial edema and of sac-yolk edema were significantly decreased in comparison to control larvae (*p* < 0.01).

In addition, high concentration CDs treatment resulted in body length alteration of the larvae. At the 96 hpf stage, the larvae in control group have significantly shorter body length than that in higher concentration (40 and 80 mg/L) groups (Table 2) (*p* < 0.01).

### 3.2. Effects of CDs on Ca^2+^-ATPase and Na^+^/K^+^-ATPase Activity of Rare Minnow Embryos/Larvae

**Figure 3:**
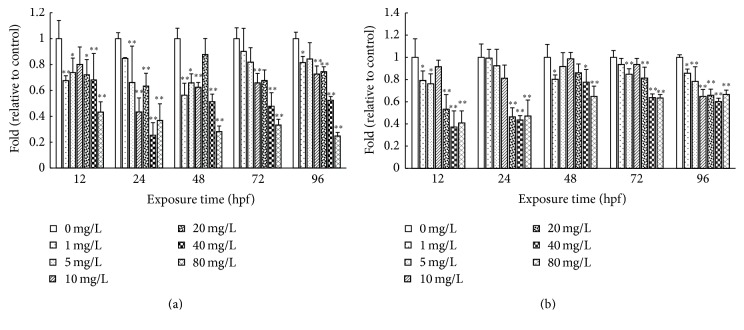
Effects of CDs on Ca^2+^-ATPase and Na^+^/K^+^-ATPase activity of rare minnow embryos/larvae. The Ca^2+^-ATPase (a) and Na^+^/K^+^-ATPase (b) activities for rare minnow embryos exposed to different concentration of CDs. Values are presented as mean ± SD (*n* = 20). Values that are significantly different from the control are indicated by asterisks (one-way ANOVA or* t*-*test*, ^*∗*^ *p* < 0.05, ^*∗∗*^ *p* < 0.01).

As shown in Figure 3, the activities of Ca^2+^-ATPase and Na^+^/K^+^-ATPase decreased in a concentration-dependent manner following exposure to CDs. In the highest concentration (80 mg/L) group, the Ca^2+^-ATPase activities were decreased to 43%, 28%, 33%, and 25% of control at the stage of 12 hpf, 48 hpf, 72 hpf, and 96 hpf, respectively. Similarly, Na^+^/K^+^-ATPase activity decreased in a concentration-dependent manner following exposure to CDs. In the highest concentration (80 mg/L) group, the Na^+^/K^+^-ATPase activities were decreased to 40%, 47%, 65%, 63%, and 67% of control at the 12 hpf, 24 hpf, 48 hpf, 72 hpf, and 96 hpf stage, respectively.

### 3.3. Development Related Genes Expression

**Figure 4:**
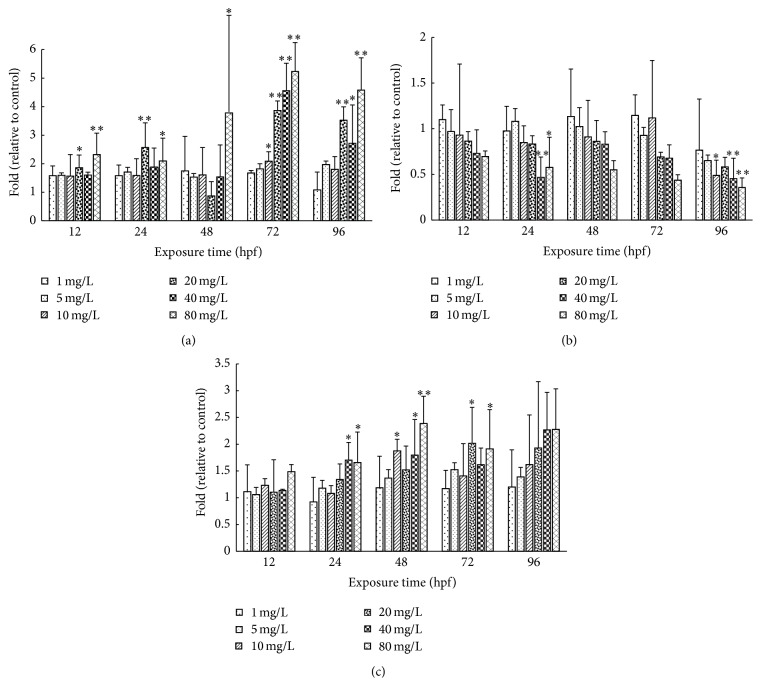
The* Mstn*,* Vezf1*, and* Wnt8a* mRNA levels for rare minnow embryos exposed to different concentration of CDs. The* Mstn *(a),* Vezf1 *(b), and* Wnt8a* (c) mRNA levels for rare minnow embryos exposed to different concentration of CDs. Values are presented as mean ± SD (*n* = 15). Values that are significantly different from the control are indicated by asterisks (one-way ANOVA or* t*-*test*, ^*∗*^ *p* < 0.05, ^*∗∗*^ *p* < 0.01).

We discuss the effects of CDs on the expression pattern of the development related genes. As shown in Figure 4,* Wnt8a*,* Vezf1*, and* Mstn* mRNA levels at different concentrations (1, 5, 10, 20, 40, and 80 mg/L) were examined with *β-actin* gene as endogenous control. The values of the mRNA levels were assessed as the fold of control. At the 12 hpf stage,* Mstn* and* Wnt8a* showed the increased expression while the* Vezf1* expression is downregulated in all concentration group.

At 24 bpf stage,* Mstn* expression was upregulated. Further, with increasing of CDs concentration, these fold changes of* Mstn* expression showed the tendency as it rose up first and then went down. Further, with increasing concentration of CDs,* Mstn* expression showed the tendency as it rose up first and then went down.* Wnt8a* expression is upregulated. In 40 and 80 mg/L group,* Wnt8a* expression is significantly elevated compared to control (*p* < 0.05). In contrast,* Vezf1* expression is downregulated as the increased CDs concentration. In 40 and 80 mg/L groups,* Vezf1* expression is significantly decreased compared to control.

At later stage (48–96 hpf),* Wnt8a* expression is upregulated as the increased CDs concentration. In response to the treatment of 80 mg/L CDs, the* Mstn* expression level increased significantly in company with the marked decrease of* Vezf1* expression at 48 hpf, 72 hpf, and 96 hpf stage (Figure 4).

### 3.4. Effects of CDs on SOD, CAT, and GPX Activities and MDA Contents of Rare Minnow Embryos/Larvae

**Figure 5:**
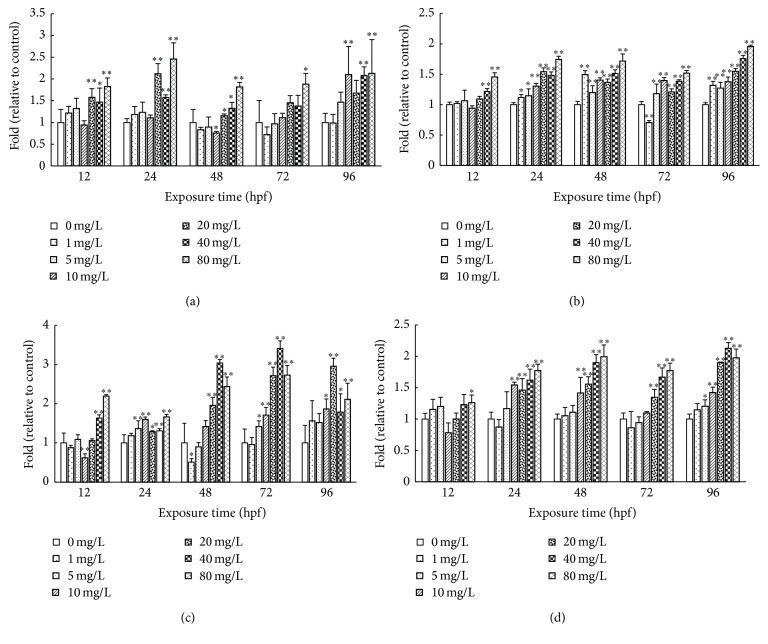
The SOD, CAT, and GPX activities and MDA contents for rare minnow embryos exposed to different concentration of CDs. The SOD (a), CAT (b), and GPX (c) activities and MDA contents (d) for rare minnow embryos exposed to different concentration of CDs. Values are presented as mean ± SD (*n* = 20). Values that are significantly different from the control are indicated by asterisks (one-way ANOVA or* t*-*test*, ^*∗*^ *p* < 0.05, ^*∗∗*^ *p* < 0.01).

We also investigated the effects of CDs on SOD, CAT, and GPX activities and MDA contents of rare minnow embryos/larvae (Figure 5). The SOD activity increased with the extending exposure duration compared with control group except at the 72 hpf stage. In the higher concentration (20, 40, and 80 mg/L) groups, significantly elevated SOD activity was detected compared with control (*p* < 0.05) (Figure 5(a)). The CAT activity alteration showed the similar pattern with that of SOD. With the extended exposure duration, significant higher CAT activity was detected accordingly (Figure 5(b)). GPX activity tended to rise up in relation to the increased CDs exposure duration. At each stage, higher CDs concentration treatment is induced sharply increase of the GPX activity (*p* < 0.05) (Figure 5(c)). CDs exposure increased the MDA content significantly compared to control at each developmental stage. As the exposure time got longer, the MDA content showed the tendency of increase which was more prominent in higher concentration groups (20, 40, and 80 mg/L) (*p* < 0.01) (Figure 5(d)).

### 3.5. DNA Damage Analysis

**Figure 6:**
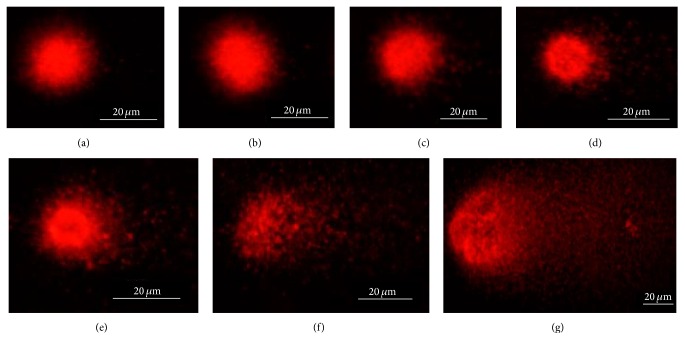
DNA damage. Effects of different concentration CDs (control (a), 1 mg/L (b), 5 mg/L (c), 10 mg/L (d), 20 mg/L (e), 40 mg/L (f), and 80 mg/L (g)) on DNA damage of rare minnow embryos/larvae cells by comet assay.

Through comet assay, we examined if the CDs exposure would induce the DNA damage in embryonic cells. The results showed that CDs exposure induces the DNA damage in cells (Figure 6). At 72 hpf stage, control cells showed uniform and regular round sized nucleus without tail (Figure 6(a)). In 1 mg/L concentration group, the cells showed oval nuclear staining without observed tail (Figure 6(b)). Elevated tail staining signal appeared in response to the increase of the CDs concentration (Figures 6(c)–6(g)).

**Figure 7:**
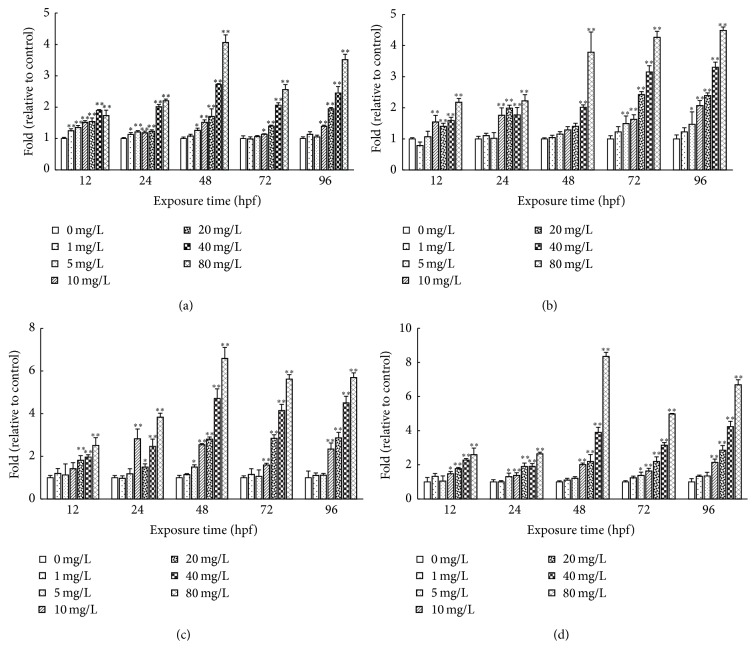
The comet tail length, tail DNA percent, tail moment, and olive tail moment for rare minnow embryos exposed to different concentration of CDs. The comet tail length (a), tail DNA percent (b), tail moment (c), and Olive tail moment (d) for rare minnow embryos exposed to different concentration of CDs. Values are presented as mean ± SD (*n* = 20). Values that are significantly different from the control are indicated by asterisks (one-way ANOVA or* t*-*test*, ^*∗*^ *p* < 0.05, ^*∗∗*^ *p* < 0.01).

Further, we tested if the tail length staining in cells is proportion to the CDs concentration with which the cells were treated. As shown in Figure 7, CDs treatment promotes the tail formation in cells. In 20, 40, and 80 mg/L group, the tail length staining is significantly stronger than control (*p* < 0.01). Except in the low concentration groups (1, 5, 10 mg/L), longer CDs exposure time promotes the length and DNA content of the comet tail. At later stage (48 hpf, 72 hpf, and 96 hpf), the length and DNA content of the comet tail increased in a concentration-dependent manner.

## 4. Discussion

### 4.1. Early Developmental Toxicity of CDs to Rare Minnow Embryos

A series of studies has shown the nanomaterials could induce the fish embryos' developmental defect among which the pericardial edema, yolk sac edema, and curvature of the spine appeared most frequently, suggesting the potential risky impacts of CDs on the natural water ecosystem.

Chen et al. have shown the abnormal condensation of embryonic eggs, pericardial edema, and curvature of the spine of zebrafish embryos/larvae following exposure to CdSe/ZnS QDs [15]. Kim and collogues showed that citrate-functionalized TiO_2_ nanoparticles caused pericardial edema, yolk sac edema, craniofacial malformation, and opaque yolk in zebrafish embryos [16]. Lv showed that nano-ZnO induced zebrafish embryonic pericardial edema, yolk sac edema, and tail malformation [17].

Consistently, our results showed the CDs exposure also induced the pericardial edema, yolk sac edema, and tail malformation in the embryos/larvae of rare minnow. These data confirm the conclusion that the nanoparticle exposure would induce the developmental defects of fish embryos/larvae. On the other, our data also suggest the pericardial edema, yolk sac edema, and tail malformation are the main consequences of nanoparticles exposure in different fish spices other than the zebrafish, implying the common potential environmental risk of the CDs and the possible dysregulated molecular mechanism of the conserved development pathway in fish embryos/larvae.

### 4.2. Molecular Mechanism Underlined the Developmental Defect Induced by the CDs Exposure

Providing energy for the active movements of Na^+^ and K^+^ across the cell membrane and the epithelia and Na^+^/K^+^-ATPase plays a central role in whole body osmoregulation, ionic transportation, muscle function, and several other membrane transportation dependent physiological processes. Previous study has reported that the activity of Na^+^/K^+^-ATPase is important for the growth and survival for the* Penaeus vannamei* in the postlarval stage [18]. Hill et al. showed the significant decreased malformation rate induced by TCDD through regulating the osmotic pressure balance of the zebrafish embryos [19], suggesting the correlation between the chemical induced hydropic malformation and osmoregulation. In our study, CDs treatment decreased the Na^+^/K^+^-ATPase activity in concentration-dependent manner (Figure 4(b)). It might block the pumping out of the Na^+^ and subsequently break the balance of Na^+^ accumulation, which eventually result in the edema and destruction of cells [20].

The hatching process of fish eggs was largely determined by the collaboration between hatching enzymes and membrane lipid peroxidation, which weakened the egg membrane along with embryos squirm [21]. In the majority of fishes, hatching enzyme is synthesized by the hatching gland distributed on the outer surface of embryo and yolk sac [22]. In our study, by means of interferometer spectrometer, we did observe that, in rare minnow, the hatching gland cells were located on the surface of yolk sac (data not shown). Thus, the defective development of the yolk sac could trigger the inactivation of hatching gland. CDs could enter into the cytoplasm by endocytosis mechanism [23]. In addition, CDs could be complexed with proteins, polysaccharide, or other biological molecules in cells, which subsequently affect the hatching enzyme activity. It could explain the lower hatching rate result from the CDs treatment (80 mg/L).

In our study, the vascular endothelial zinc finger (*Vezf1*) expression level decreases with the increased CDs concentration (Figure 5(b)).* Vezf1* participates in the molecular pathways that control early blood vessel development.* Vezf1* may play an important role in the endothelial lineage determination and embryonic vasculogenesis and angiogenesis at later stages [24]. Silencing* Vezf1* on chicken embryo in vivo suggested that* Vezf1* is important for the development of blood vessel and heart [25]. Decreased expression levels of* Vezf1* influenced the function of cardiovascular system during early development. Eventually, the heart physiological compensation promotes the increased heart rate and SV-BA distance of the embryos in 10~80 mg/L concentration group (Table 2).

In our study, the Ca^2+^-ATPase activities decreased with the increased CDs concentration and exposure duration (Figure 3(a)). The Ca^2+^-ATPase plays a crucial role in maintaining the intracellular concentration of Ca^2+^[26]. Inhibition of the activity of Ca^2+^-ATPase leads to the intracellular accumulation of Ca^2+^ which induces the skeletal developmental defect. Myostatin (*Mstn*) and* Wnt8a* expressions were observed to be upregulated significantly in response to the CDs treatments in a concentration-dependent manner (Figures 4(a) and 4(c)).* Mstn* belongs to the transforming growth factor-b (TGF-b) family and has been identified as an important negative regulator of muscle development [27].

MOD mediated muscle development is tightly regulated by the TGF-*β* pathway. As the important transcriptional regulator of MOD, Mstn could be recruited by the SMAD proteins to the regulatory sequences of MOD which account for the retarded muscle growth [28]. Wnt8a is a key factor in Wnt/*β*-catenin signaling pathway for the body axis extending [29]. Wnt8a initially locates at the bottom area of the animal pole of fertilized eggs which activate the classical Wnt/*β*-catenin signaling through translocating to the destined microtube region, contributing to the curvature of the spine or tail within the development progression [30]. Downregulated* Wnt8a* results in lacking of the tail shaft structure in zebrafish embryos/larvae [31]. The significant increased expression of* Wnt8a* indicates the inhibited body axis extension. Taken together, on one hand, both of the* Wnt8a* expression upregulation and the inhibited Ca^2+^-ATPase activity promote the skeletal development and, on the other hand, increased* Mstn* expression blocks the muscle development which could explain the observed tail and spinal curvatures in this study.

It is noteworthy that the detailed mechanism underlined the CDs induced alteration of the target gene expression is poorly understood. Previous studies have shown that the CDs could be complexed with both double strand DNA (dsDNA) [32] and cellular functional proteins [9]. These results provide the probability that the CDs could affect the transcription of target gene through either cis- or transregulation. Accordingly, in our study, we reasoned that there are two possible mechanisms either working separately or together to alter the development related gene expression induced by CDs exposure: (1) the CDs might bind to the cis-elements which block the binding sites of transcriptional factors. (2) The CDs might be complexed with and dysregulated the transcriptional factors directly. However, the further study is needed to clarify this issue in the future.

### 4.3. Oxidative Stress and DNA Damage

Oxidative DNA damage and the inflammatory response by far are the two major involved mechanisms concerning the biological toxicity of the nanomaterials [33, 34]. In normal physiological condition, the endogenous antioxidant enzymes combat the reactive oxygen species (ROS) and prevent the oxidative damage to the organism. In fact, fish can combat the oxidative stress with the enzyme system consisting of SOD, CAT, and GPX. SOD convert superoxide anions (O_2_^−^) into H_2_O_2_ and then the enzymes of CAT and GPX catalyze the reaction to digest H_2_O_2_ into H_2_O and O_2_^−^ [35]. Upon exogenous stress, abnormal ROS accumulation would break this balance and generate cellular oxidative damage.

Environmental pollutants go into the body and produce active oxygen free radicals through a series of metabolic conversions. If they are not cleared promptly, the balance will be destroyed and cause organisms oxidative damage [36]. LPO can be defined as the oxidative damage of cell membrane lipids and has been used extensively as a biomarker of oxidative stress in vivo [37]. As one of the main products of cell membrane LPO, MDA level has been regarded as the indicator of the LPO level [38].

In the present study, the activities of SOD were increased following CDs treatments (Figure 5(a)), suggesting the occurrence of the oxidative stress. SOD act first to scavenge the O_2_^−^; then antioxidant system becomes active to defense and synchronizes the activity of SOD with that of CAT. These results are in agreement with the previous study [39] in which the correlations between SOD and CAT were found in the mussel* Mytilus galloprovincialis* upon the stress introduced by copper nanoparticles exposure. Moreover, due to the accumulating H_2_O_2_ generated from O_2_^−^ by SOD, the CAT turn active quickly to keep pace with SOD (Figure 5(b)). Also, the GPX activity is induced by the higher intensity of stress result from higher CDs concentration (Figure 5(c)).

In sum, in rare minnow, the antioxidant enzyme system consists of SOD, CAT, and GPX combined together that act against oxidative stress and keep the MDA content in a certain range in lower CDs concentration group. However, long time of CDs exposure in 20, 40, and 80 mg/L concentration groups breaks the organism antioxidant capacity and results in the increase of the MDA content (Figure 5(d)), indicating that CDs exposure induced the oxidative stress to the embryo. Consequently, the defective development of rare minnow embryo appeared.

With the increased CDs concentration and extended exposure time, serious DNA damage of rare minnow embryos/larvae cells was detected (Figures 6 and 7). It could be the reason for higher malformation and mortality rate. Further increase of the CDs concentration results in more serious DNA damage induced by the ROS attacking nucleotides. The damaged DNA cannot be repaired in time; thus the malformation rate and mortality rate increased significantly [40].

## 5. Conclusion

1. In summary, the higher concentrations of CDs have significant development toxicity on rare minnow embryos which could be characterized as decreased spontaneous movements and body length, increased heart rate, and pericardial edema, yolk sac edema and tail/spinal curvature, various morphological malformations, and decreased hatching rate.
2. The underlying mechanism of this developmental toxicity appears to be related to the generation of oxidative stress, repressed DNA repair efficacy, and altered development related gene expression.

## Figures and Tables

**Table 1:** Primers sequences used for real-time PCR.

Gene	Forward primers	Reverse primers
*β-Actin*	CCCCATTGAGCACGGTATTG	GGGAGCCTCTGTGAGCAGGA
*Wnt8a*	CCAAAGGCTTACCTCACA	AACCCAACCACGACCC
*Vezf1*	GTGGCGGGCATCCTCACCAC	GCCGCACATCTCACAGCCGT
*Mstn*	CACCGCCTTTGCAACAACTT	CCGATCTACTTGAACGATGG

**Table 2:** Effects of CDs on behavior and morphological parameter measurement of rare minnow embryos/larvae.

Indicator	0 mg/L	1 mg/L	5 mg/L	10 mg/L	20 mg/L	40 mg/L	80 mg/L
Spontaneous movements of 36 hpf (bends/30 seconds)	11.17 ± 2.32	9.50 ± 1.52	10.17 ± 1.47	8.17 ± 2.79^*∗∗*^	6.83 ± 1.94^*∗∗*^	7.83 ± 1.17^*∗∗*^	9.17 ± 1.17
Heart rate of 60 hpf (bends/30 seconds)	67.00 ± 2.45	64.50 ± 2.88	60.67 ± 4.17^*∗*^	64.00 ± 4.24	75.50 ± 3.27^*∗∗*^	70.67 ± 5.74	71.00 ± 3.58
Malformation rate (%)	5.00 ± 1.00	6.67 ± 1.73	8.33 ± 1.73	13.30 ± 1.73^*∗∗*^	26.67 ± 4.58^*∗∗*^	25.00 ± 3.00^*∗∗*^	43.33 ± 4.58^*∗∗*^
SV-BA (×10^2^ *μ*m)	1.90 ± 0.19	2.07 ± 0.27	2.15 ± 0.28	2.70 ± 0.45^*∗∗*^	3.13 ± 0.26^*∗∗*^	3.05 ± 0.47^*∗∗*^	3.32 ± 0.26^*∗∗*^
Area of pericardial edema (×10^4^ *μ*m^2^)	2.48 ± 0.53	2.60 ± 0.65	2.97 ± 0.32	4.05 ± 0.67^*∗∗*^	5.43 ± 0.64^*∗∗*^	5.50 ± 0.97^*∗∗*^	5.55 ± 0.75^*∗∗*^
Area of sac-yolk edema (×10^5^ *μ*m^2^)	2.44 ± 0.10	2.56 ± 0.10	2.42 ± 0.22	2.83 ± 0.16^*∗∗*^	3.17 ± 0.23^*∗∗*^	3.49 ± 0.27^*∗∗*^	3.41 ± 0.27^*∗∗*^
Body length (mm)	3.11 ± 0.21	3.13 ± 0.18	3.16 ± 0.11	3.03 ± 0.08	2.96 ± 0.14	2.82 ± 0.20^*∗∗*^	2.80 ± 0.18^*∗∗*^

Values are presented as mean ± SD. Spontaneous movements, heart rate, SV-BA, area of pericardial edema, and area of sac-yolk edema (*n* = 6); body length (*n* = 7) and malformation rate (*n* = 60). Values that are significantly different from the control are indicated by asterisks (one-way ANOVA or *t*-*test*,^*∗*^ *p* < 0.05, ^*∗∗*^ *p*< 0.01).
